# Age-related accumulation of toxic metals in the human locus ceruleus

**DOI:** 10.1371/journal.pone.0203627

**Published:** 2018-09-19

**Authors:** Roger Pamphlett, David P. Bishop, Stephen Kum Jew, Philip A. Doble

**Affiliations:** 1 Discipline of Pathology, Sydney Medical School, Brain and Mind Centre, The University of Sydney, New South Wales, Australia; 2 Department of Neuropathology, Royal Prince Alfred Hospital, Sydney, New South Wales, Australia; 3 Elemental Bio-imaging Facility, University of Technology, Sydney, New South Wales, Australia; Chinese Academy of Sciences, CHINA

## Abstract

Damage to the locus ceruleus has been implicated in the pathogenesis of a number of neurological conditions. Locus ceruleus neurons accumulate toxic metals such as mercury selectively, however, the presence of toxic metals in locus ceruleus neurons of people of different ages, and with a variety of disorders, is not known. To demonstrate at what age toxic metals are first detectable in the locus ceruleus, and to evaluate whether their presence is more common in certain clinicopathological conditions, we looked for these metals in 228 locus ceruleus samples. Samples were taken at coronial autopsies from individuals with a wide range of ages, pre-existing conditions and causes of death. Paraffin sections of pons containing the locus ceruleus were stained with silver nitrate autometallography, which indicates inorganic mercury, silver and bismuth within cells (termed autometallography-detected toxic metals, or AMG^™^). No locus ceruleus AMG neurons were seen in 38 individuals aged under 20 years. 47% of the 190 adults (ie, aged 20 years and over) had AMG locus ceruleus neurons. The proportion of adults with locus ceruleus AMG neurons increased during aging, except for a decreased proportion in the 90-plus years age group. No differences were found in the proportions of locus ceruleus AMG neurons between groups with different neurological, psychiatric, or other clinicopathological conditions, or among various causes of death. Elemental analysis with laser ablation-inductively coupled plasma-mass spectrometry was used to cross-validate the metals detected by AMG, by looking for silver, gold, bismuth, cadmium, chromium, iron, mercury, nickel, and lead in the locus ceruleus of ten individuals. This confirmed the presence of mercury in locus ceruleus samples containing AMG neurons, and showed cadmium, silver, lead, iron, and nickel in the locus ceruleus of some individuals. In conclusion, toxic metals stained by AMG (most likely inorganic mercury) appear in locus ceruleus neurons in early adult life. About half of adults in this study had locus ceruleus neurons containing inorganic mercury, and elemental analysis found a range of other toxic metals in the locus ceruleus. Locus ceruleus inorganic mercury increased during aging, except for a decrease in advanced age, but was not found more often in any single clinicopathological condition or cause of death.

## Introduction

The locus ceruleus supplies noradrenaline to the central nervous system, by which it influences neuronal and glial function and maintains the blood-brain barrier [1]. The locus ceruleus plays a role in cognition [2], response to stressors [3], neuroinflammation [4], and in the control of impulsivity [5]. Damage to the locus ceruleus with loss of its neurons has been implicated in autopsy studies of conditions as diverse as aging [6], Alzheimer disease [2], Parkinson disease [1], depression [7], bipolar disorder [8], schizophrenia [9] and the sudden infant death syndrome [10]. Recent structural and functional imaging studies have confirmed a loss of locus ceruleus neurons during life in aging and Alzheimer and Parkinson diseases, and have shed light on how decreased noradrenaline leads to changes in locus ceruleus connections that can explain cognitive changes in these conditions [11,12].

The underlying cause of damage to the locus ceruleus that contributes to these various disorders remains unknown, though it has been hypothesised that a selective uptake of environmental toxicants into the locus ceruleus could be the culprit, possibly combined with genetic susceptibility to these toxicants [13]. Mercury is a toxicant suspected to play a part in disorders such as Alzheimer disease [14], Parkinson disease [15] and amyotrophic lateral sclerosis [16], and mercury is taken up selectively by the locus ceruleus [17]. Mercury is an attractive candidate for these disorders since humans worldwide are exposed to mercury, particularly via fish consumption and mercury-containing dental restorations [18,19], and mercury has actions that are injurious to cells, the immune system and genes [19].

Although toxic metals have been found in locus ceruleus neurons in patients with Alzheimer disease [20] and amyotrophic lateral sclerosis [17,21], it is not clear to what extent these metals are present in the locus ceruleus in other disorders, or at what age they start appearing in the locus ceruleus. It is difficult to assess the contribution of toxic metals to neurodegenerative disorders because neuronal loss is severe by the time brain tissue becomes available via autopsy. We therefore examined locus ceruleus neurons for toxic metals in people who had had Australian coronial autopsies (similar to the USA medical examiner system). This gave us the opportunity to estimate at what age toxic metals first become detectable in the locus ceruleus, to examine toxins in the locus ceruleus in a wide range of clinicopathological disorders and causes of death, and to look for reasons for the variability of toxic metal uptake into the locus ceruleus between individuals. The histochemical technique of autometallography was used to look for toxic metals; this demonstrates inorganic mercury, but can also show silver and bismuth. To validate which autometallography-detected metals were likely to be present, we used laser ablation-inductively coupled plasma-mass spectrometry to look for mercury, silver, and bismuth, as well as other toxic metals, in the locus ceruleus in a subset of individuals.

## Materials and methods

### Study design

The prevalence of AMG^™^-detected toxic metals in the human locus ceruleus has previously been estimated in amyotrophic lateral sclerosis [21], Alzheimer disease [20] and autism [22], and in small numbers of older adults without neurological disease [20,21]. These samples, however, were not suitable to estimate the prevalence of locus ceruleus toxic metals within a more general population of varying ages. With the decline in numbers of hospital autopsies, it is difficult to get access to autopsy brain tissue from people with a wide range of ages who have died from a variety of conditions, so we sourced tissue samples from coronial autopsies. Australian coroners investigate sudden and unexpected deaths to determine the identity of the deceased, and circumstances and medical causes of death, and an autopsy is ordered by the coroner to ensure that balanced, accurate findings regarding the cause of death can be delivered (http://www.coroners.justice.nsw.gov.au). Our primary aim was to obtain locus ceruleus samples for analysis of toxic metals from at least 10 people in each decade of life, with a mixture of clincopathological conditions and causes of death within each decade. Our second aim was to see if any groups of people numbering 10 or more, with various clinicopathological conditions or causes of death (cases), were more likely to have toxic metals in their locus ceruleus than groups with no known pre-mortem conditions, or undetermined and sudden causes of death (controls). A third aim was to look for any common factors among people who had high levels of locus ceruleus toxic metals to try to understand why these people had a particular predilection to accumulate toxic metals in their locus ceruleus.

Autopsy reports between 1997 and 2014 from the New South Wales Department of Forensic Medicine were examined to find individuals in whom the locus ceruleus had been examined microscopically. To ensure that a varied sample of the coronial population was studied, these reports were filtered to select a variety of clinicopathological conditions, such as a history of incarceration, cancer, neurodegenerative disorder (eg, Alzheimer or Parkinson disease) or psychiatric disorder (eg, depression or schizophrenia). Further samples were chosen from individuals who had died from sudden non-medical causes of death, eg, drowning, suicide or trauma. Cases were collected until ethics-approved numbers for age decades, individual clinicopathological conditions, and causes of death were reached. Neurodegenerative disorders were confirmed on neuropathological examination of brains. Brain and body weights, and body heights, were recorded when available. A total of 228 individuals were studied, with numbers of individuals in successive age ranges being 28 (0–9 years), 10 (10–19 years), 27 (20–29 years), 45 (30–39 years), 32 (40–49 years), 11 (50–59 years), 18 (60–69 years), 16 (70–79 years), 14 (80–89 years) and 27 (90-plus years). Adults were defined as those aged 20 years and over.

### Ethics

This study, "The role of the locus ceruleus in disorders of the nervous system" (X14-029), was approved by the Human Research Committee, Sydney Local Health District (RPAH Zone), and by the Office of the New South Wales Coroner. The institutional review board waived the need for written informed consent from relatives of individuals studied since this was a de-identified retrospective study of autopsy tissue.

### Tissue sectioning and autometallography

Seven μm paraffin sections of pons containing the locus ceruleus from all 228 individuals, and in 65 of these individuals (including 39 adults) the midbrain containing the substantia nigra, were stained for inorganic mercury, silver and bismuth bound to sulphides or selenides, with silver nitrate autometallography (AMG), an amplification technique that can detect only a few atoms of these metals within tissues, including the human brain [23,24]. Briefly, sections were placed in physical developer containing 50% gum arabic, citrate buffer, hydroquinone and silver nitrate at 26°C for 80 min in the dark then washed in 5% sodium thiosulphate to remove unbound silver. Sections were counterstained with mercury-free Improved Harris hematoxylin and viewed with bright-field microscopy. Each staining run included a positive control section of mouse spinal cord with motor neuron cell bodies containing mercury following an intraperitoneal injection of 2 μg/g mercuric chloride [25]. Sections were also stained with hematoxylin and eosin, and hematoxylin alone, to assess general pathology. Selected pons sections were immunostained, after AMG staining, with mouse anti-human CD31 monoclonal antibody (Dako) at 1:100 visualised with Bond Polymer Refine Red to assess the capillary density within the locus ceruleus, and rabbit anti-human glial fibrillary acidic protein polyclonal antibody (Dako) at 1:2000 visualised with diaminobenzidine tetrahydrochloride to see if AMG was present in locus ceruleus astrocytes.

Numbers of locus ceruleus AMG neurons were counted using a 10x10 eyepiece grid viewed at 400x magnification, with right and lower exclusion margins, and stepped sequentially throughout one transverse section of the locus ceruleus [21]. An AMG-stained neuron (ie, an “AMG neuron”) was defined as any locus ceruleus neuron with a minimum diameter of 26 μm (the length of one grid square) that contained 10 or more black AMG grains. The proportion of AMG neurons within the locus ceruleus was then classified as either grade 0 (none), grade I (1–10%), grade II (11–50%) or grade III (>50%). To assess the biological significance of toxic metals in the locus ceruleus, individuals with locus ceruleus samples containing 0–10% of AMG neurons (grades 0 and I) were deemed to be in a “low category” as regards toxic metals, while those with >10% of AMG neurons (grades II and III) were deemed to be in a “high category”.

### Laser ablation-inductively coupled plasma-mass spectrometry

Seven μm paraffin sections of ten pons samples containing the locus ceruleus (seven male and three female, six with negative AMG and four with grade III AMG) were deparaffinised and subjected to LA-ICP-MS analysis [26] for the three metals stained by AMG (inorganic mercury, silver and bismuth), other toxic metals (gold, cadmium, chromium and lead) and the trace metals iron and nickel. Imaging was performed using a New Wave Research UP213 (Kennelec Scientific, Mitcham, Victoria, Australia) Nd:YAG laser system with a two-volume large format cell connected to an Agilent 7500cx ICP-MS (Agilent Technologies, Mulgrave, Victoria, Australia). The ICP-MS was fitted with ‘s’ lenses for enhanced sensitivity and argon was used as the carrier gas. LA-ICP-MS conditions were optimised on NIST 612 Trace Element in Glass CRM and the sample was ablated with a 55 μm spot size and a scan speed of 220 μm s-1 at a frequency of 20 Hz. The data were collated into a single image file using in-house developed software and visualised using Paraview. Limits of detection using LA-ICP-MS are estimated to be between 0.05 and 0.81 μg per g [27].

### Statistical analyses

GraphPad Prism 7 software was used for unpaired t-tests to look for differences between means of normally distributed continuous variables, chi-square 2x2 analyses with Fisher’s exact tests to look for associations between two categorical variables when all cell numbers were ≥5, and chi-square tests for trend to analyse locus ceruleus toxic metals in relation to age. Significance was assessed at the 0.05 level.

## Results

### General pathology

**Fig 1:**
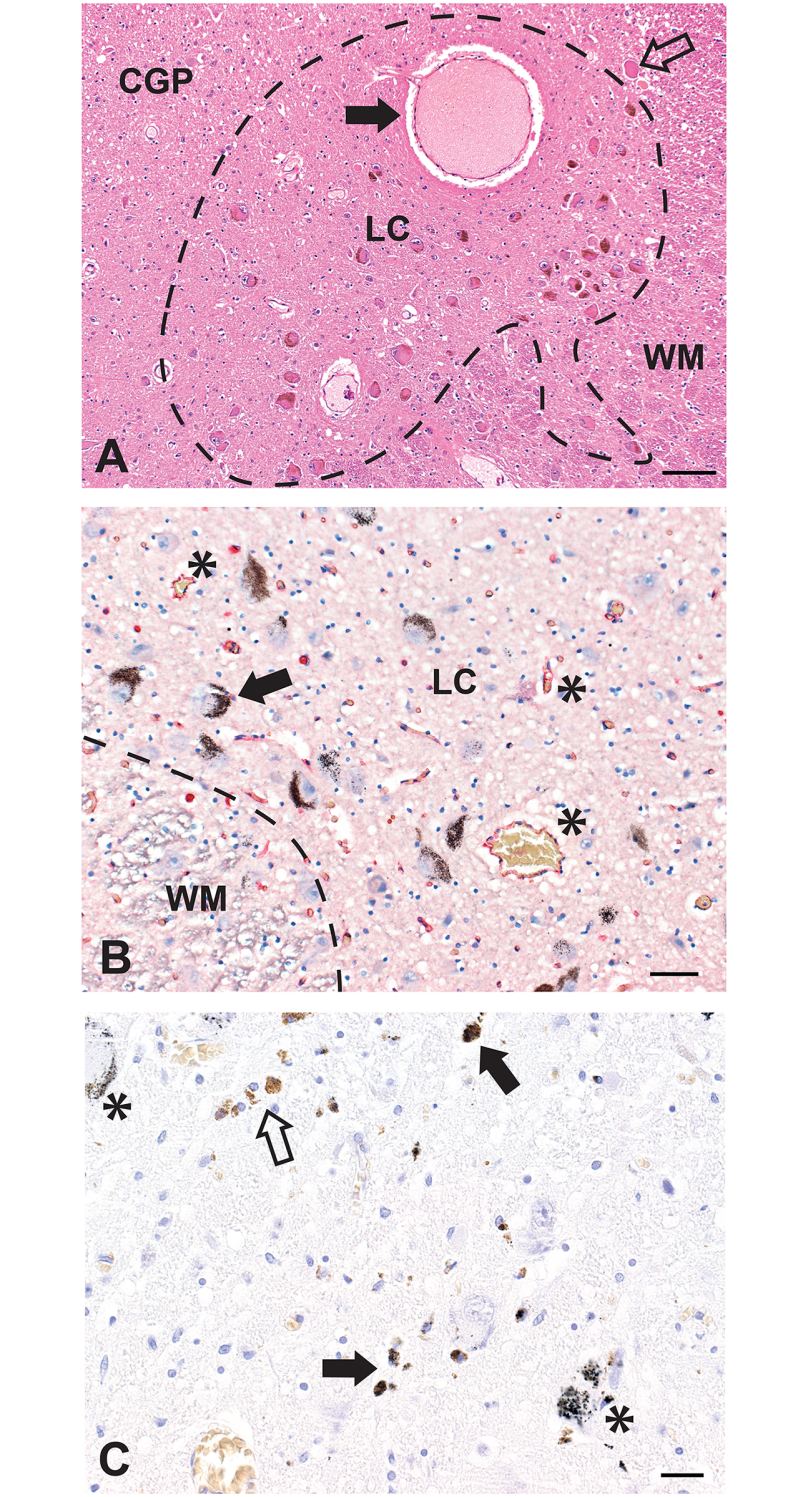
Normal appearance and pathology of the locus ceruleus. (A) The locus ceruleus (LC, outlined) contains numerous, mostly neuromelanin-containing, neuronal cell bodies. White matter (WM) is present at the right, and the central grey of the pons (CGP) at the left. A large thin-walled blood vessel (closed arrow), probably a post-capillary venule, is seen within the locus ceruleus. A mesencephalic trigeminal neuron is seen in the upper right corner (open arrow). Hematoxylin and eosin, Bar = 100 μm. (B) The capillary density in the locus ceruleus is slightly increased, compared to adjacent white matter (WM), as seen by the red-immunostained capillary endothelial cells (some asterisked). Black AMG grains are present in several locus ceruleus neurons (one with arrow). AMG/hematoxylin/CD31, Bar = 50 μm. (C) This locus ceruleus has a reduced density of neurons. Free and macrophage-bound neuromelanin originating from destroyed locus ceruleus neurons is prominent, with this pigment having either AMG (closed arrow) or no AMG (open arrow). Some remaining locus ceruleus neurons either with (asterisks) or without AMG are seen. AMG/hematoxylin, Bar = 20 μm.

Locus ceruleus neurons, most containing neuromelanin, were found in the posterolateral aspect of the rostral pons. At least one large thin-walled blood vessel was usually present within or adjacent to the locus ceruleus (Fig 1A). Capillary density in the locus ceruleus appeared to be slightly increased compared to surrounding regions (Fig 1A and 1B). In individuals with Alzheimer or Parkinson diseases a marked loss of locus ceruleus neurons was seen, with numerous collections of macrophage-bound and free neuromelanin pigment either with or without AMG (Fig 1C). In older individuals, some neuromelanin was often present in the neuropil in the absence of obvious locus ceruleus neuronal loss. In conditions other than neurodegenerations, locus ceruleus neuronal numbers and densities appeared to be within normal limits, though no formal quantitation was undertaken since the pons had not been sampled at the same transverse level in each person.

### Autometallography

#### Locus ceruleus neuronal AMG

**Fig 2:**
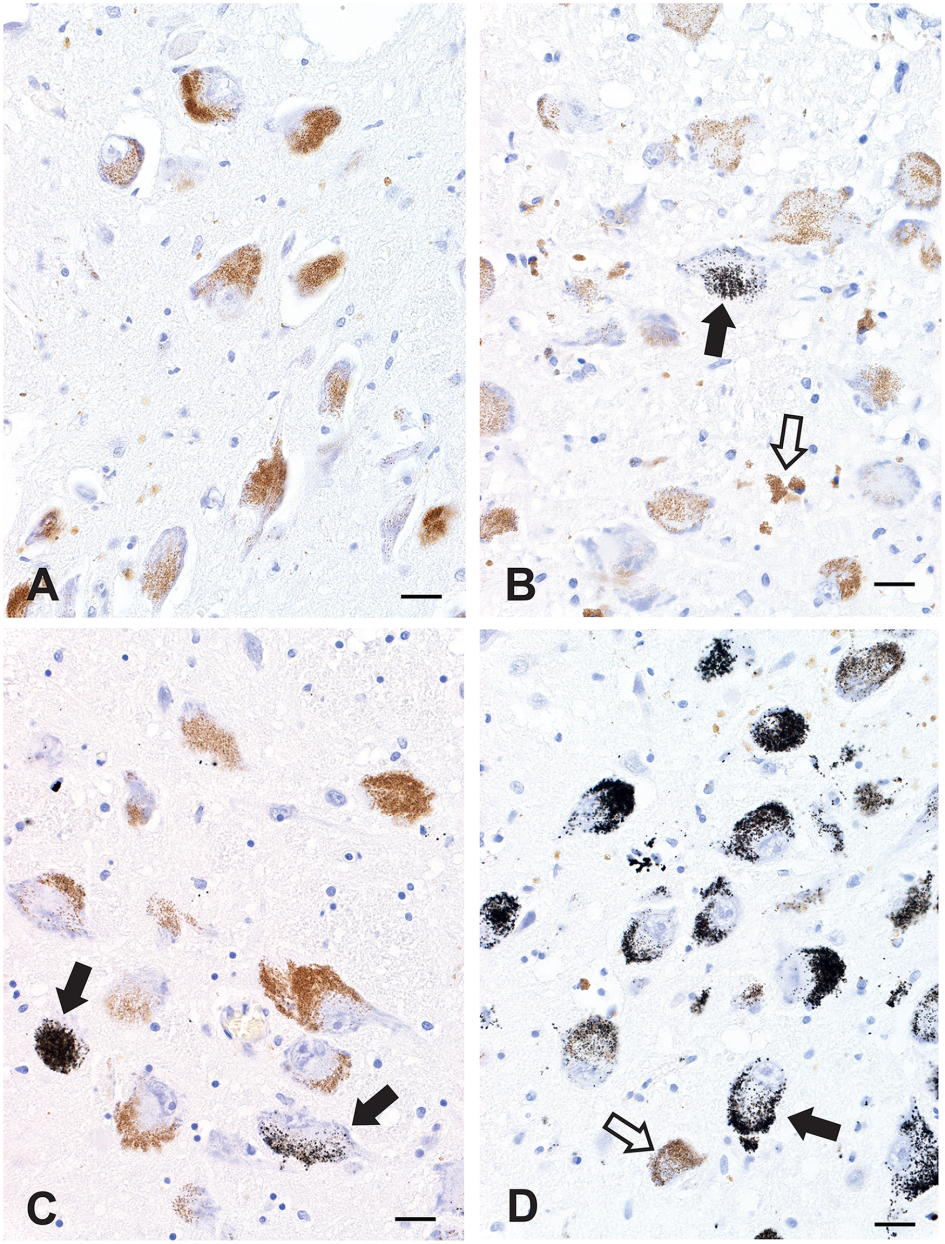
Grades of AMG in the locus ceruleus. (A) Grade 0. No black AMG grains are seen in these locus ceruleus neurons that contain yellow-brown cytoplasmic neuromelanin granules. (B) Grade I. One (ie, fewer than 10%) of these locus ceruleus neurons (closed arrow) contains AMG. Free and macrophage-bound neuromelanin pigment (without AMG, open arrow) is seen in the neuropil. (C) Grade II. Two locus ceruleus neurons (arrows, 10–50%) stain with AMG. (D) Grade III. More than 50% of locus ceruleus AMG neurons are present. The intensity of staining varies between neurons, with a heavily-stained neuron (closed arrow) adjacent to a non-stained neuron (open arrow). AMG is also present in scattered free and macrophage-bound neuromelanin. All figures AMG/hematoxylin, Bars = 20 μm.

Normal locus ceruleus neurons without AMG (grade 0) contained yellow-brown granules of neuromelanin (Fig 2A). In grade I, fewer than 10% of locus ceruleus AMG neurons were seen (Fig 2B); in grade II, 11–50% of locus ceruleus neurons stained with AMG (Fig 2C); in grade III, more than 50% of locus ceruleus neurons stained with AMG (Fig 2D). The intensity of AMG varied between neurons of the same individual, and admixtures of non-stained, lightly-stained and heavily-stained neurons were present in most people (Fig 2C). Both neuromelanin-containing and non-pigmented locus ceruleus neurons contained AMG, though when neurons were stained heavily with AMG it was not possible to tell if underlying neuromelanin was present.

#### Locus ceruleus capillary-associated AMG

**Fig 3:**
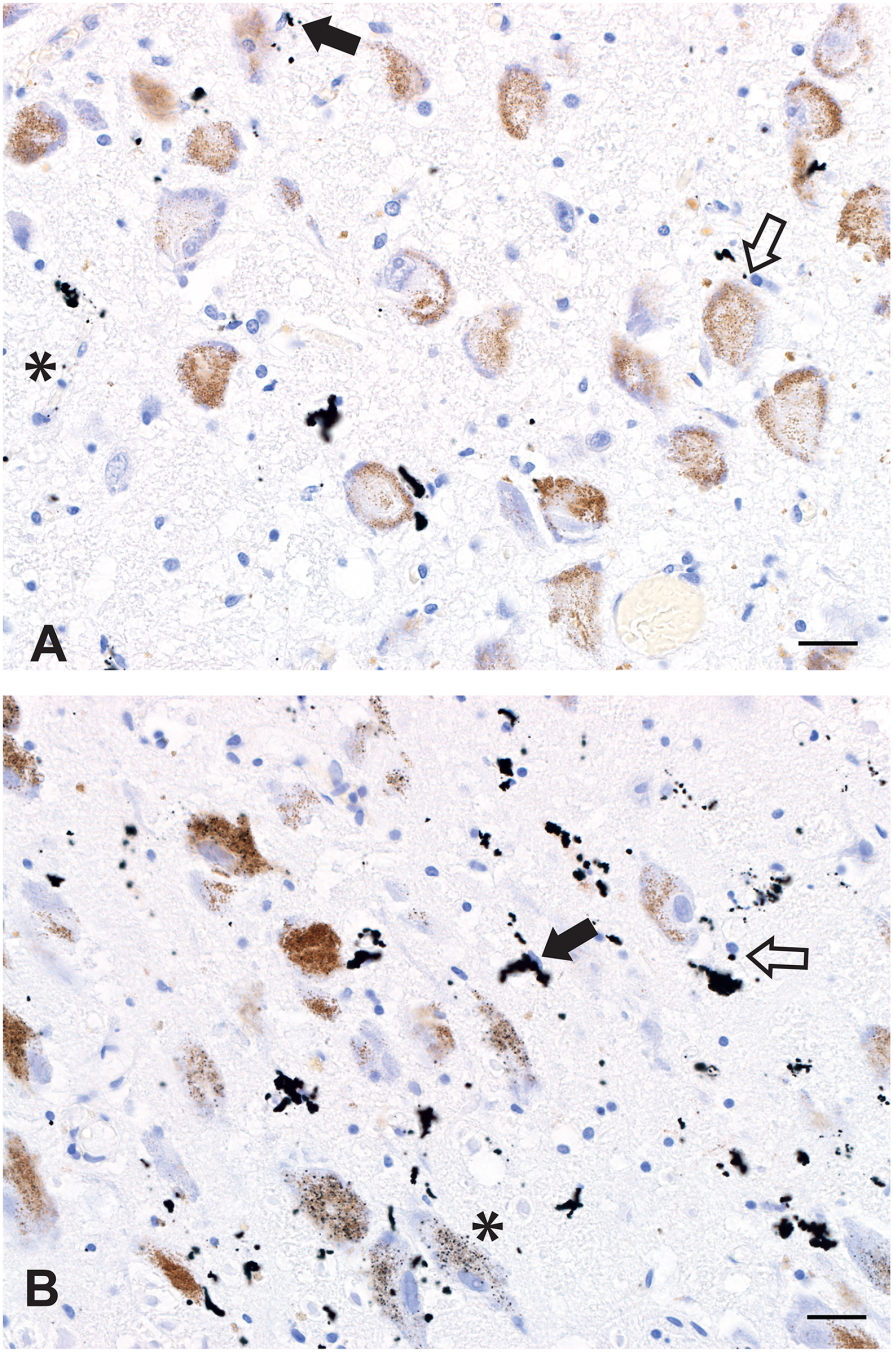
Capillary-associated AMG in the locus ceruleus. (A) Small punctate or larger mulberry-shaped black AMG deposits are present in and around capillary walls. Small deposits can be identified adjacent to oval (endothelial cell, closed arrow) or round (pericyte, open arrow) nuclei, or in capillary walls (asterisk). No neuronal AMG is present. (B) More extensive mulberry AMG deposits are seen in a locus ceruleus with numerous AMG neurons (one with asterisk). Small AMG deposits can be seen adjacent to endothelial cell (closed arrow) or pericyte (open arrow) nuclei. Both figures AMG/hematoxylin, Bar = 20 μm.

Small dense particulate AMG was seen associated with capillaries in about 20% of adult locus ceruleus samples. These particles were adjacent to cell nuclei that had either oval (probably endothelial cell) or round (probably pericyte) profiles (Fig 3A and 3B). Some AMG was aggregated into mulberry-like clusters. The presence of this AMG did not correlate closely with the presence of locus ceruleus AMG neurons, ie, it could be present when no locus ceruleus AMG neurons were seen, and absent when locus ceruleus AMG neurons were abundant. No other region of the pons contained these particles.

#### AMG in other cells

No AMG was seen within glial fibrillary acid protein-immunostained astrocytes in the locus ceruleus. Occasional scattered neurons in the *pontine reticular formation* and *dorsal raphe nuclei* contained AMG, usually in sections in which locus ceruleus grade III AMG was present. No AMG was seen in other pontine neurons, eg, in the mesencephalic nucleus of the trigeminal nerve immediately dorsolateral to the locus ceruleus (Fig 1A). One individual with Parkinson disease had *widespread neuronal staining* with AMG, which also affected locus ceruleus and *substantia nigra* neurons. No substantia nigra neurons contained AMG in any of the other 38 adults in whom the midbrain was sampled (despite 16 of these having locus ceruleus AMG neurons), nor in any of the 26 individuals aged less than 20 years. Perivascular neurons in pontine basal nuclei contained non-specific (ie, seen on all stains) multiple small dark-brown birefringent particulate cytoplasmic bodies in some sections, of undetermined nature.

#### Age and locus ceruleus AMG

**Fig 4:**
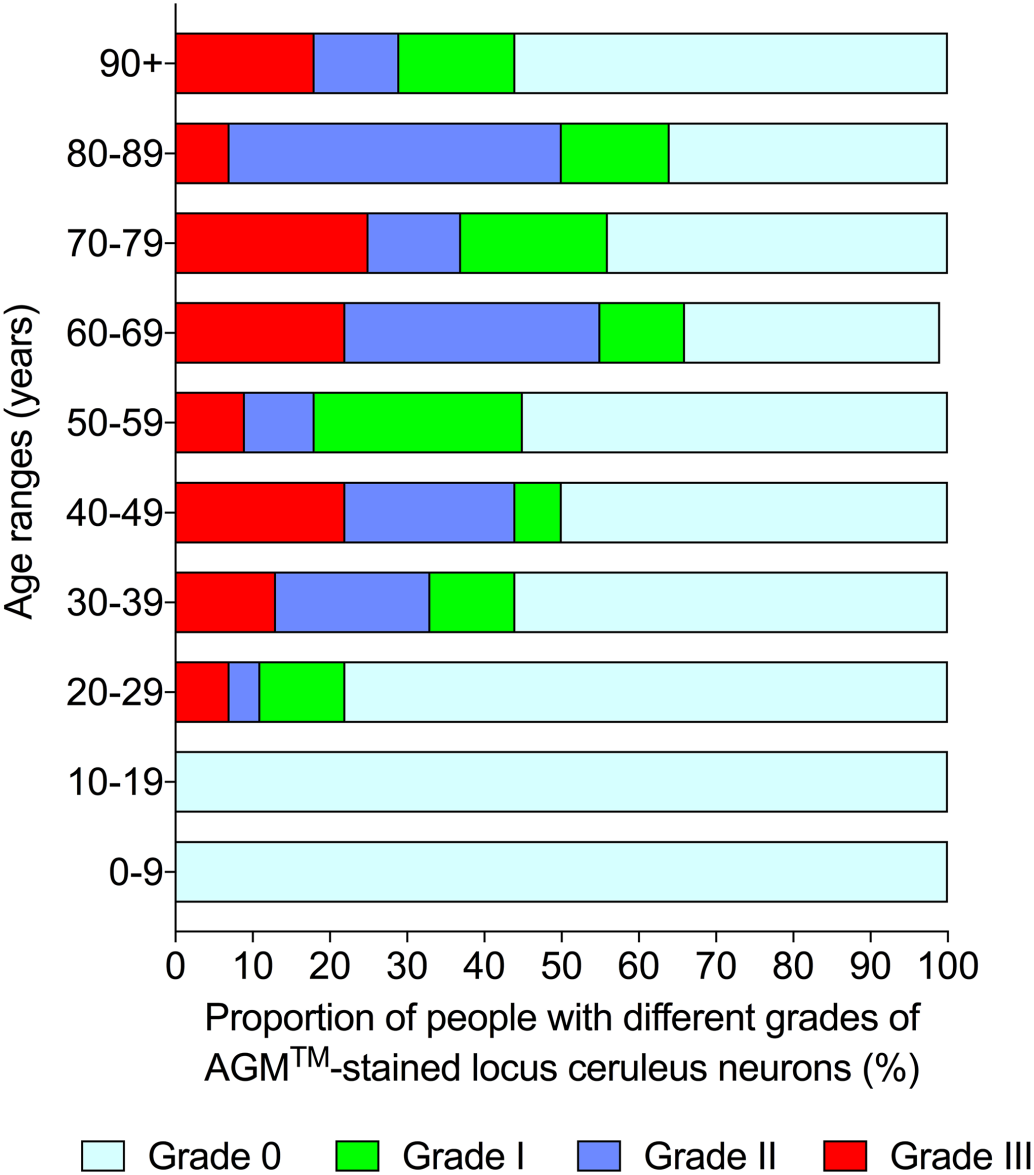
Grades of locus ceruleus AMG and increasing age. A mixture of all four grades of locus ceruleus AMG was found in all adult age decades.

No locus ceruleus AMG neurons were seen in the 38 infants and adolescents under the age of 20 years. In the 190 adults aged ≥20 years, a total of 53% had grade 0, 13% grade I, 18% grade II, and 16% grade III AMG. All four grades of AMG were present in each adult age decade (Fig 4).

**Fig 5:**
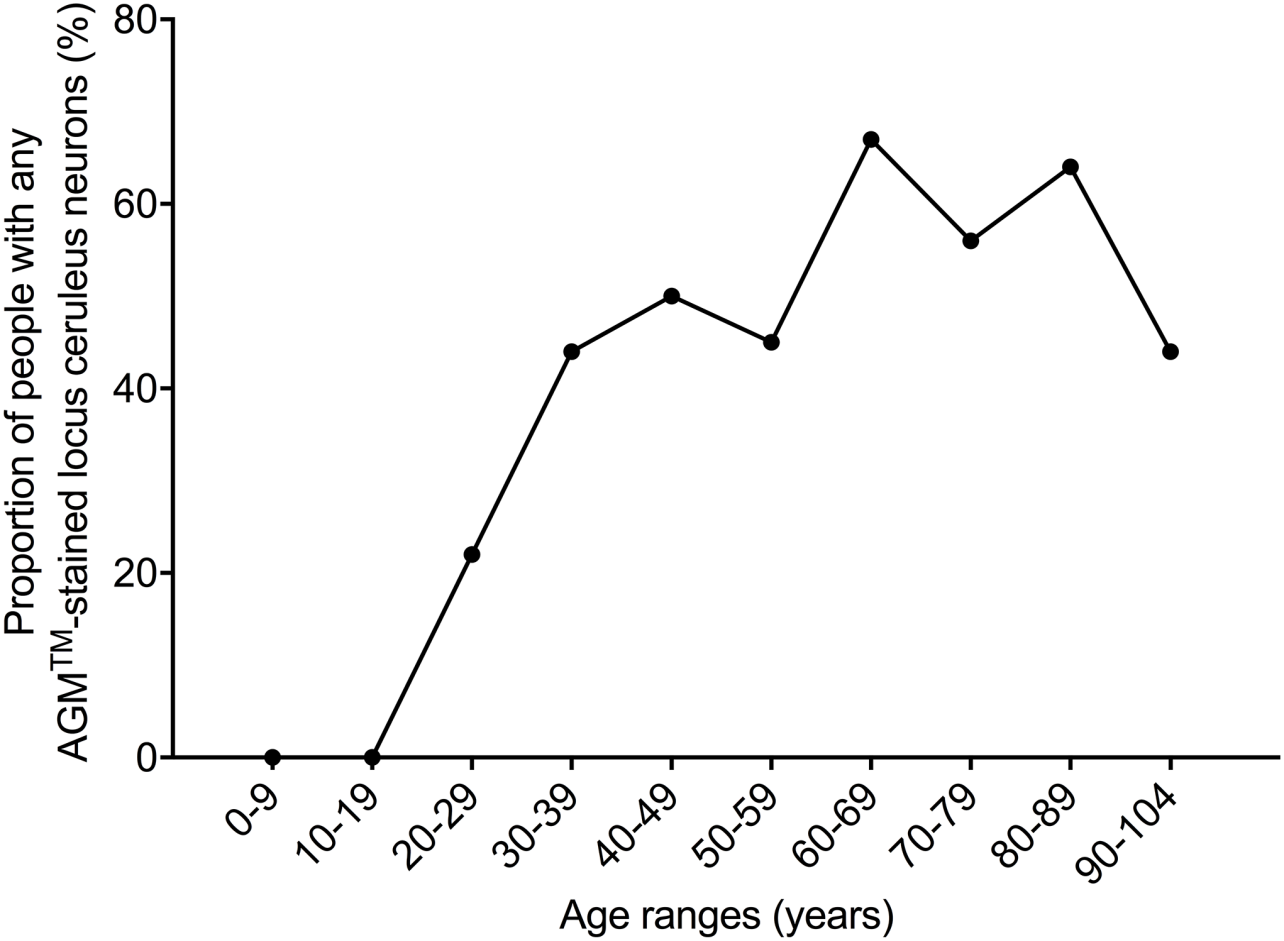
Proportions of people with any locus ceruleus AMG and increasing age. The proportion of people with any locus ceruleus neuronal AMG increased from the third decade onwards, but with a decrease in the final 90-plus years age range.

The proportion of people with any locus ceruleus neuronal AMG increased from none between the ages of 0 and 19 years, to a low level in the third decade (22%), then plateaued at a moderate level between the ages of 30 and 60 years (44–50%), followed by a higher plateau between the ages of 60 and 90 years (56–67%), before falling back to a moderate level from the age of 90 years on (44%) (Fig 5, chi-square trend for aging 21.9, p<0.0001).

#### Clinicopathological diagnoses and locus ceruleus AMG

Comparisons between low and high category locus ceruleus AMG in adult locus ceruleus samples in different clinicopathological groups can be seen in Table 1. Only the combined neurodegeneration group had a significantly higher proportion of high level AMG compared to a control group with no known pre-mortem conditions, but this could have been because the mean age of the neurodegeneration group (77 years) was higher than that of the control group (55 years). Individuals with locus ceruleus grade III AMG neurons were present in all clinicopathological conditions when case numbers were ≥10. The combined psychiatric disorder group was similar to the control group as regards low and high locus ceruleus AMG levels.

**Table 1:** Clinicopathological conditions of adults with low (grades 0 and I) or high (grades II and III) categories and grade III alone, of locus ceruleus AMG.

Clinicopathological condition	Low category locus ceruleus AMG *N* (%)	High category locus ceruleus AMG *N* (%)	Chi-square p	Grade III locus ceruleus AMG *N*
Anorexia nervosa	0 (0)	1 (100)	-	1
Bipolar disorder	4 (44)	5 (56)	-	3
Cancer	3 (75)	1 (25)	-	0
Crime	23 (85)	4 (15)	-	1
Dementia—Alzheimer	11 (65)	6 (35)	0.75	2
Dementia—FTD	0 (0)	1 (100)	-	0
Dementia—Lewy body	4 (80)	1 (20)	-	0
Dementia—NOS	3 (75)	1 (25)	-	1
Depression	6 (67)	3 (33)	-	2
Down syndrome	1 (100)	0 (0)	-	0
Epilepsy	1 (33)	2 (67)	-	0
Huntington disease	0 (0)	1 (100)	-	1
MSA	1 (25)	3 (75)	-	1
Myotonic dystrophy	0 (0)	1 (100)	-	1
Neurodegenerative disorders	30 (51)	29 (49)	0.045	10
None known (Controls)	32 (71)	13 (29)	NA	6
Obesity	3 (100)	0 (0)	-	0
Parkinson disease	11 (55)	9 (45)	0.25	5
PSP	0 (0)	1 (100)	-	0
PTSD	2 (100)	0 (0)	-	0
Psychiatric disorders	28	19	0.28	
Schizophrenia	18 (62)	11 (38)	0.45	5
Stroke	1 (50)	1 (50)	-	1
Substance abuse	1 (100)	0 (0)	-	0

-: cell number less than 5; AMG: toxic metal autometallography; Controls: used as control for chi-square testing; FTD: frontotemporal dementia; MSA: multiple system atrophy; NA: not applicable; Neurodegenerative disorders: sum of Dementia, Huntington disease, Myotonic dystrophy, MSA, Parkinson disease and PSP; NOS: not otherwise specified; PSP: progressive supranuclear palsy; Psychiatric disorders: sum of Bipolar disorder, Depression and Schizophrenia; PTSD: post-traumatic stress disorder.

No pre-mortem clinicopathological conditions were identified in the 28 individuals aged <10 years (14 male, 14 female). Two of the 10 individuals aged 10–19 years (7 male, 3 female) had a history of epilepsy.

#### Causes of death and locus ceruleus AMG

Comparisons between low and high category locus ceruleus AMG among various causes of death in adults can be seen in Table 2. No statistically-significant differences in numbers of people with these levels of AMG within the locus ceruleus was found for any groups of causes of death, using the Undetermined and sudden causes of death as a control group. Neither the combined medical nor combined non-medical causes of death groups showed differences in locus ceruleus AMG compared to the control group. Individuals with grade III AMG were found in all causes of death groups when case numbers were ≥10.

**Table 2:** Causes of death in adults who had low (grades 0 and I) or high (grades II and III) categories, or grade III alone, of locus ceruleus AMG.

Cause of death	Low category locus ceruleus AMG *N* (%)	High category locus ceruleus AMG *N* (%)	Chi-square p	Grade III locus ceruleus AMG *N*
Asphyxia	4 (44)	5 (56)	-	0
Cancer	2 (67)	1 (33)	-	0
Cardiovascular	15 (54)	13 (46)	0.35	7
Cirrhosis	1 (100)	0 (0)	-	0
Controls	37 (65)	20 (35)	NA	10
Drown	14 (78)	4 (22)	-	3
Drug overdose	13 (68)	6 (32)	>0.99	3
Homicide	1 (25)	3 (75)	-	1
Hypothermia	1 (100)	0 (0)	-	0
Infection	9 (64)	5 (36)	>0.99	4
Medical conditions	33 (63)	19 (37)	>0.99	11
Non-medical conditions	85 (68)	40 (32)	0.73	18
Respiratory	1 (100)	0 (0)	-	0
Stroke	4 (100)	0 (0)	-	0
SUDEP	2 (67)	1 (33)	-	0
Suicide	36 (73)	13 (27)	0.40	5
Trauma	16 (64)	9 (36)	-	5
Undernutrition	0 (0)	1 (100)	-	1
Undetermined	6 (60)	4 (40)	-	1

-: cell number less than 5; AMG: toxic metal autometallography; Controls: combined Drown, Homicide, Trauma and Undetermined used as control for chi-square testing; Medical conditions: sum of cancer, cardiovascular, cirrhosis, infection, respiratory, stroke and SUDEP; NA: not applicable; Non-medical conditions: all except Medical conditions and Undetermined; SUPDEP: sudden unidentified death in epilepsy.

Causes of death in the 28 individuals aged <10 years were the sudden infant death syndrome (*N* = 20), trauma (*N* = 5) and drowning (*N* = 3). In the 10 individuals aged 10–19 years causes of death were trauma (*N* = 4), undetermined (*N* = 2) and one each of drowning, drug overdose, suicide and respiratory.

#### Gender and locus ceruleus AMG

Adult males (*N* = 124, mean 49.5 y, SD 22.1 y, range 20–100 y) were on average younger than adult females (*N* = 66, mean 64.1 y, SD 268 y, range 20–104 y) (difference between means 14.6 y, 95% CI 7.4–21.8 y, p <0.0001). The proportions of adults with different grades of locus ceruleus AMG did not differ between males and females on Fisher’s exact testing. Proportions for males and females respectively were grade 0: 53.2% and 53.0% (*p* = 0.99), grade I: 15.3% and 7.6% (*p* = 0.17), grade II: 19.4% and 16.7% (*p* = 0.70) and grade III: 12.1% and 22.7% (*p* = 0.06). The tendency for Grade III to be more common in females was probably because there were more older females.

#### Brain weight, body mass index and locus ceruleus AMG

Brain weights of adults did not differ significantly between low category (*N* = 125, mean 1366 g, SD 185 g, range 830–1880 g) and high category (*N* = 65, mean 1351 g, SD 181 g, range 1002–1790 g) locus ceruleus AMG groups (*p* = 0.58). Adult body mass index did not differ significantly between low category (*N* = 117, mean 25.8, SD 7.5, range 10–52) and high category (*N* = 64, mean 25.9, SD 8.7, range 9–61) locus ceruleus AMG groups (*p* = 0.92). There are fewer numbers in these groups since body heights were not always available to calculate body mass index.

### Laser ablation-inductively coupled plasma-mass spectrometry (LA-ICP-MS)

**Fig 6:**
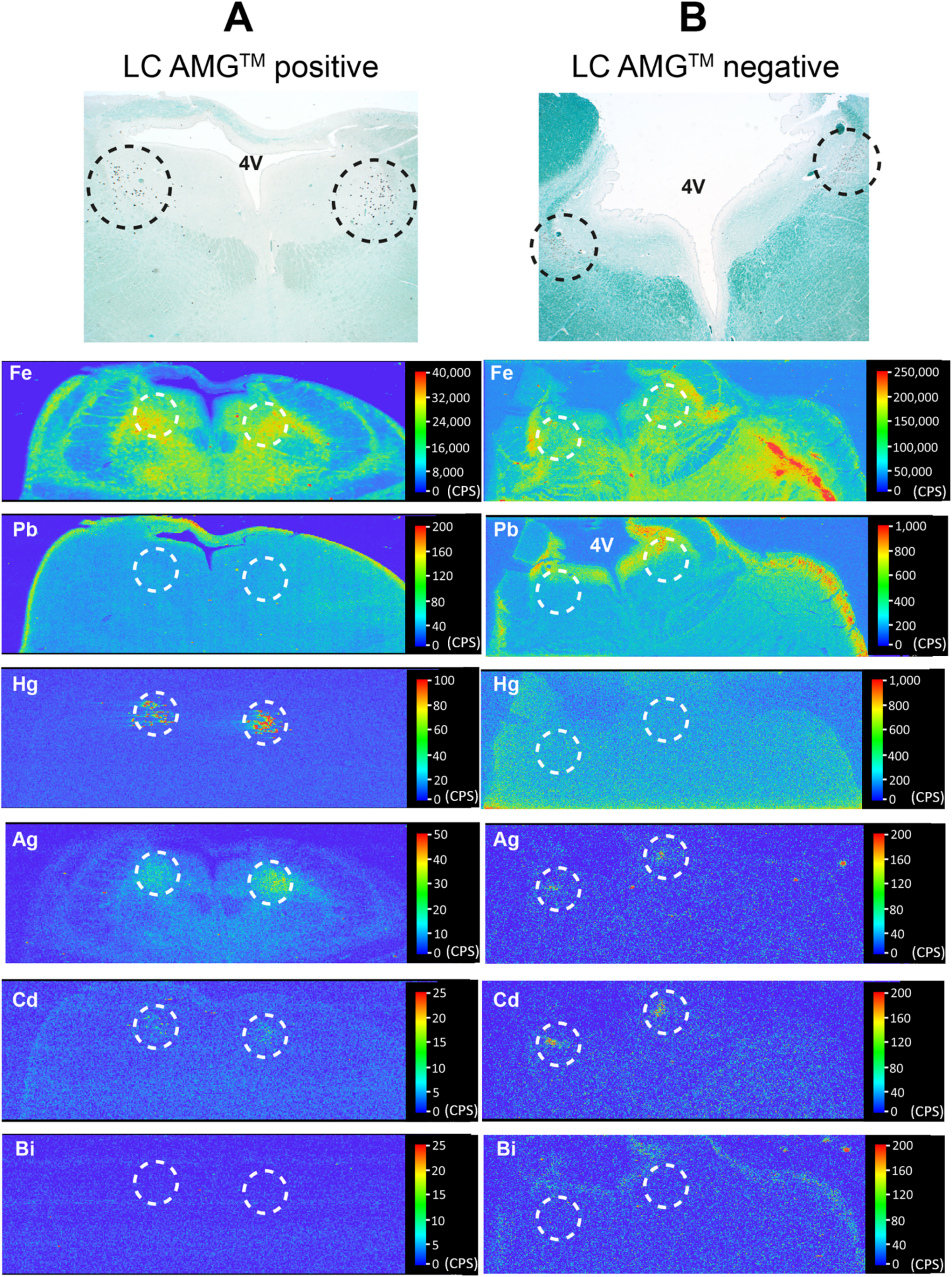
LA-ICP-MS of the posterior pons of individuals with either positive or negative locus ceruleus AMG. Sections stained with AMG and Luxol-fast blue for myelin (at the top of the figure) show the position of the locus ceruleus (within dashed circles) in the posterior pons. (A) A 20–29 year-old with Huntington disease and locus ceruleus AMG (Table 3) has mercury, silver and cadmium in the locus ceruleus (situated within the dashed circles) but no iron, lead or bismuth. Iron and nickel are present in the grey matter, and lead in the subpial pons. (B) A 40–49 year-old with schizophrenia and no locus ceruleus AMG neurons (Table 3) has silver and cadmium in the locus ceruleus, but no mercury, iron, lead or bismuth above background levels. Iron and nickel are present in the grey matter, and lead in the subpial and periventricular pons. The linear collection of iron (red, on the right side of the pons) is in the distribution of a blood vessel and is probably iron in red blood cells. 4V: fourth ventricle. CPS: counts per second.

Mercury was detected on LA-ICP-MS in the locus ceruleus of all four individuals in whom locus ceruleus neurons were stained with AMG (Table 3, Fig 6A). No LA-ICP-MS mercury was found in five of the six individuals with no locus ceruleus AMG. In one individual, LA-ICP-MS mercury was detected in the locus ceruleus in the absence of AMG, indicating this was likely to be organic mercury (which is not detectable on AMG). LA-ICP-MS-detected inorganic mercury was therefore the only metal consistently correlated with AMG (Table 3). Cadmium (Cd) was present in the locus ceruleus of all ten people (Fig 6, Table 3). Both iron (Fe) and nickel (Ni) were seen in the grey matter of the posterior pons (Fig 6), and in three individuals in the locus ceruleus in amounts above background (Table 3). Silver (Ag) was found in the locus ceruleus of people both with and without AMG (Fig 6), suggesting this silver is bound to proteins and so not visible on AMG. In three people, silver was also seen in the grey matter anterolateral to the silver-containing locus ceruleus (Fig 6A). Lead (Pb) was seen in the pons of all individuals, either in the grey matter or widespread in the grey and white matter. In four people, lead was predominantly localised to the subpial surfaces and periventricular region of the pons (Fig 6B), suggesting uptake from the cerebrospinal fluid. Bismuth (Bi), the third metal that can be detected by AMG, was not seen in the pons of any individuals (Fig 6), and neither was gold (Au) nor chromium (Cr). In the two individuals who had locus ceruleus capillary-associated AMG, no pattern of elemental accumulation could explain the nature of these deposits (Table 3).

**Table 3:** LA-ICP-MS analyses of metals in the posterior pons of individuals either with or without locus ceruleus AMG.

Decade y	Condition	AMG	Hg	Ag	Bi	Au	Cd	Cr	Fe	Ni	Pb
30–39	Bipolar	Positive	+	-	-	-	+	-	- G	- G	- G
>90	Aged	Positive	+	-	-	-	+	-	- G	- G	- G
20–29	Huntington	Positive	+	+	-	-	+	-	- G	- G	- S
60–69	Drown	Positive	+	+	-	-	+	-	+ G	+ G	+ G
50–59	Drown	Negative	-	-	-	-	+	-	- G	- G	- G
50–59	Schizophrenia	Negative	-	+	-	-	+	-	- G	- G	- W
60–69	Drown	Negative ^	+	+	-	-	+	-	+ G	+ G	- S
40–49	Drown	Negative	-	+	-	-	+	-	- G	+ G	- W
40–49	Schizophrenia	Negative ^	-	+	-	-	+	-	- G	- G	+ S
40–49	Schizophrenia	Negative	-	+	-	-	+	-	+ G	- G	- S

Positive: Grade III AMG in locus ceruleus, Negative: no AMG in locus ceruleus, ^: locus ceruleus capillary-associated AMG; +: metal present in locus ceruleus, -: metal not present in locus ceruleus, G: metal present in grey matter, S: metal present in subpial and periventricular pons, W: metal widespread in grey and white matter.

## Discussion

Key findings in this study are that toxic metals (most likely inorganic mercury) were detected in about half of adults from a mixed clinicopathological and cause of death population. The toxic metals started to appear in locus ceruleus neurons after the age of 20 years, and the proportion of people with locus ceruleus toxic metals increased until a decrease in late old age. No clinicopathological condition or cause of death was statistically associated with the presence of locus ceruleus AMG-demonstrable toxic metals. Elemental analysis of a subset of individuals confirmed that inorganic mercury was likely to be the metal responsible for the AMG staining. For the purposes of discussion, it will therefore be assumed that in most individuals the toxic metal demonstrated by AMG was inorganic mercury bound to sulfides and selenides, this being the form of mercury recognised by AMG.

An increase in inorganic mercury in the locus ceruleus with aging could be due to accumulated mercury from mercury-containing dental amalgam restorations, occupational exposure to inorganic mercury, as well as exposure to organic mercury since methylmercury from fish, the commonest human source of mercury, is slowly demethylated in the human brain to inorganic mercury [19,28]. Inorganic mercury appears to be the proximate toxic mercury species in the CNS [29], and the slow accumulation of brain inorganic mercury over years or decades [30] could account for the late-life onset of neurodegenerative diseases: these could be precipitated when the concentration of inorganic mercury in locus ceruleus neurons reached a neurotoxic tipping point [31]. However, deleterious effects of inorganic mercury in the locus ceruleus would be most likely only if other factors predisposing to neurotoxicity were present, such as genetic susceptibilities [32], selenium deficiency [33] and synergistic interactions between mercury and other toxic metals [19,34].

The proportion of people with locus ceruleus inorganic mercury declined in the 90-plus years age group, decreasing from its heights in the previous three decades. This may be because those who survived to this advanced age were exposed to insignificant amounts of mercury throughout their lives and therefore avoided the many diseases associated with mercury toxicity [35]. The lower amount of locus ceruleus inorganic mercury in later life may therefore be the reason for the reduced incidence in advanced age of Alzheimer disease [36], Parkinson disease [37] and amyotrophic lateral sclerosis [38], all disorders that have been associated with exposure to mercury [39]. Less toxic metal content of the locus ceruleus in advanced age could also underlie the plateau of human mortality in later life that has been described, with death rates levelling off from 105 years and beyond [40], since people who had not been exposed to toxic metals in early life could be expected to have fewer causes of mortality when older.

Older adults with a high cognitive reserve have higher locus ceruleus neuroimaging signal intensities than those with a low reserve [41], and arguably less of a tendency to develop Alzheimer disease and early death, so they may have lower levels of locus ceruleus toxic metals. On the other hand, some autopsy studies report a continued loss of locus ceruleus neurons even in the oldest old [6], so the relationship between aging and changes in locus ceruleus histology and signal intensity remains unclear [42]. An increased neuromelanin signal in the locus ceruleus in middle age may be due to increased neuromelanin production as a defensive manoeuvre to bind toxic metals [43]. On the other hand, a decrease in neuromelanin signal intensity in later life could be due to a loss of locus ceruleus cells caused by prolonged oxidative damage from, for example, accumulated metals [44]. A potential confounding factor is that toxic metals such as mercury can reside within neurons in sufficient amounts to cause axonal shrinkage, but not to destroy the cell body [25], implying that mercury could reduce locus ceruleus noradrenaline output without causing cell loss.

Inorganic mercury was taken up by the locus ceruleus in only some people in each of the different clinicopathological and cause of death groups. A clue as to why this is so may be that various stressors upregulate locus ceruleus activity for prolonged periods of time [45]. So, in people who are subject to psychological stress at the same time as being exposed to toxic metals, stress-activated locus ceruleus neurons could accumulate these metals. This would also explain the apparently random accumulation of inorganic mercury within locus ceruleus neurons, since if a subset of locus ceruleus neurons were activated by a stressor at the time of toxic metal exposure, they alone would be the ones to take up the toxic metal. It is therefore of interest that many people who had dense AMG staining of the locus ceruleus also had conditions in which high levels of psychological stress have been described. These include stressful conditions such as dementia [46], Parkinson disease [47] and schizophrenia [3], as well as stress-inducing conditions in which there is no reason to suspect that toxic metals plays a primary role, such as Huntington disease [48] and anorexia nervosa [49]. We unfortunately have no way of knowing what stressors these individuals were subjected to during life, but this concept of stress-induced toxin uptake could be explored with animal models.

It is not clear why the locus ceruleus accumulates toxic metals selectively. Rhesus monkeys have an extensive capillary blood supply to the locus ceruleus [50], but in our study human locus ceruleus capillary density was only slightly increased compared to surrounding tissue. Locus ceruleus neurons within individuals varied markedly in inorganic mercury content, indicating that a high blood flow to the entire locus ceruleus is unlikely to be a major factor underlying this variable uptake. The locus ceruleus is close to the fourth ventricle and so could be exposed to toxins in the cerebrospinal fluid [2], but we found that locus ceruleus neurons close to the fourth ventricle did contain more inorganic mercury than those at a greater distance from the ventricle. Mercury could also employ a noradrenaline-associated transporter to enter locus ceruleus neurons, in a similar way the renal tubule mercury transporter operates [51]. Toxic metals could also enter locus ceruleus neurons via retrograde uptake from the extensive axonal connections these neurons have with brain microvessels [13]. Finally, mercury could accumulate in locus ceruleus neurons by binding to neuromelanin [52] and not be available for elimination via the glymphatic or other pathways. However, inorganic mercury was only rarely seen in neuromelanin-containing cells of the substantia nigra in our study, so the presence of neuromelanin alone is unlikely to account for the large amounts of mercury seen in the locus ceruleus.

Several lines of evidence link neurodegenerative disorders with mercury and the locus ceruleus. Locus ceruleus neuronal loss is marked in many neurodegenerative disorders in which associated losses of noradrenaline have been described [53]. The locus ceruleus is involved early in Alzheimer disease [54] and people with Alzheimer disease have more inorganic mercury in their locus ceruleus than controls [20]; furthermore, hyperphosphorylated tau is often found in the human locus ceruleus [54], and mercury causes tau hyperphosphorylation [55] and aggregation [56]. The normal locus ceruleus may protect the substantia nigra from damage [57] so the locus ceruleus may be the primary site of damage in Parkinson disease [58]. Rotenone has been implicated in Parkinson disease; it is of interest that the locus ceruleus is more sensitive to the effects of rotenone than the substantia nigra [59], and that rotenone demethylates methylmercury to more neurotoxic inorganic mercury [60]. Abnormalities in the locus ceruleus have been described in multiple sclerosis [61], with mercury being suspected to be an environmental trigger for the disease [62]. Most multiple sclerosis patients present for the first time in early adulthood [63], the age at which we first saw inorganic mercury appearing in the locus ceruleus.

Inorganic mercury in our study did not appear to be markedly more frequent in the locus ceruleus of people with neurodegenerative conditions. However, locus ceruleus neuronal loss is severe in Alzheimer and Parkinson diseases, and the remaining neurons available for autopsy study could be the ones that never contained inorganic mercury (a "survivor" effect).

Damage to the locus ceruleus has been implicated in psychiatric conditions [64], but we did not find more inorganic mercury in the locus ceruleus of people with depression, bipolar disorder or schizophrenia, either for each individual condition, or when psychiatric diagnoses were grouped together. We also found no locus ceruleus inorganic mercury in infants who died from the sudden infant death syndrome, in which a defect in arousal secondary to locus ceruleus damage has been postulated [10].

The finding of inorganic mercury in the locus ceruleus may be a clue as to why incidences of several neurological disorders have been either increasing or decreasing over time. This may be because human exposure to the two major sources of environmental mercury, fish consumption and mercury-containing dental restorations, are going in different directions. Rising atmospheric mercury, mostly from burning fossil fuels [65], is converted by aquatic microorganisms into methylmercury, which is increasingly bioaccumulated by fish consumed by humans [66]. The rising incidences described for amyotrophic lateral sclerosis [67], Parkinson disease [68] and multiple sclerosis [69] could be linked to this increasing exposure to mercury, originating from atmospheric sources. On the other hand, the incidence of Alzheimer disease appears to be decreasing in a number of countries [70], in parallel with falling exposure to inorganic mercury from reduced numbers of mercury-containing dental restorations [71]. Further epidemiological studies of mercury exposure in these disorders may cast more light on this issue.

Mercury within the locus ceruleus could affect noradrenaline output in two opposite ways. First, oxidative stress caused by mercury [19] could decrease noradrenaline output, with downstream consequences in particular for Alzheimer and Parkinson diseases [72]. Second, mercury could paradoxically *increase* noradrenaline output from the locus ceruleus by binding to and damaging cysteine-rich enzymes that normally deactivate noradrenaline, such as coenzyme S-adenosylmethionine; this is a mechanism proposed for an increase in noradrenaline output from mercury-containing adrenal medulla ganglion cells that can cause arterial hypertension [73]. Increased noradrenaline output from the locus ceruleus following mercury exposure could therefore also have a bearing on other conditions suspected to involve an increase in noradrenaline output, such as the post-traumatic stress disorder [74].

Elemental analysis of a subset of individuals showed that other neurotoxic metals apart from mercury can be taken up selectively by the locus ceruleus. Cadmium was found in the locus ceruleus of all ten individuals examined, which may not be surprising since cadmium exposure is common from widespread environmental sources [75], including cigarette smoking [76]. Cadmium in cigarette smoke taken up by the locus ceruleus may therefore be the reason smoking is linked to the risk of amyotrophic lateral sclerosis [77], Alzheimer disease [78] and multiple sclerosis [79]. The finding that lead (Pb) in two individuals was localised to subpial and periventricular surfaces exposed to cerebrospinal fluid is of interest, given the finding that inorganic mercury can also be concentrated in subpial regions and may play a part in the pathogenesis of the subpial and periventricular demyelinating plaques of multiple sclerosis [80]. Lead was also seen concentrated in the locus ceruleus in two individuals. Silver was present in the locus ceruleus of six of the ten individuals studied; silver is found in dental amalgam restorations (together with mercury) and is increasingly used in nanoparticles for commercial and medical applications [81]. The silver in the locus ceruleus was not reliably picked up by AMG in our study and so was most likely to be in a protein-bound form. The trace elements iron and nickel were found in grey matter regions of the pons, and three individuals had increases in iron and nickel in their locus ceruleus. Iron has been linked to a number of neurodegenerative disorders [82]. Iron levels increase in the brain throughout life [83] but we could not estimate an age effect since we did not have quantitative measures of these elements. Finally, the finding of multiple toxic metals within the locus ceruleus may have pathogenetic implications since harmful synergistic interactions between toxic metals, as well as between toxic and essential metals, have been described [19,34].

LA-ICP-MS did no show any consistent pattern of metals in two individuals who had locus ceruleus capillary-associated AMG. Greater numbers of cases with capillary-associated AMG will need to be analysed for more metals to assess the chemical makeup of these collections.

Limitations of the study are: (1) Full medical histories and complete results of investigations were not available, since these were not hospital autopsies. However, major pre-mortem diagnoses and medications were listed, and full autopsy pathology results were available from all individuals. (2) The study aimed primarily to look at age as a factor in the uptake of toxins into the locus ceruleus, so numbers of people with specific diagnoses or causes of death were often limited. Further investigations of locus ceruleus toxic metals within specific disease subgroups would best be performed using tissue from disease-specific brain banks. (3) We did not quantitate the metals found within the locus ceruleus, but this is planned as a future project. (4) Coronial autopsy populations, aimed largely at investigating unnatural deaths, are unlikely to precisely replicate conditions found in general living populations. We attempted to minimise the differences by studying people with a wide range of disorders, as well as those without known medical conditions who died suddenly and unexpectedly. Of note, the proportion of people in this study who had locus ceruleus AMG staining was similar to that of smaller age-matched brain donor populations who had no neurological disorders [20,21]. (5) The study was biased towards younger men, since more men than woman have coronial autopsies to investigate unnatural deaths. The older women in our study tended to have more grade III locus ceruleus staining than the younger men, but the other grades of staining showed no gender effect, so gender would be unlikely to affect the overall age-related findings of the study.

## Conclusions

Toxic metals in the locus ceruleus can be found at autopsy in about half of an adult population with diverse clinicopathological conditions and causes of death. The toxic metal detected by autometallography is most likely to be inorganic mercury, which has the potential to damage locus ceruleus neurons and affect noradrenaline output to the brain. This has potential consequences in particular for neurodegenerative and psychiatric disorders. The proportion of locus ceruleus neurons containing toxic metals appears to increase throughout adulthood, except for a decrease in advanced age. This may underlie why some neurodegenerative disorders have reduced incidences at advanced ages, and why mortality rates appear to be stable in the older old. No particular medical condition or cause of death was associated with the amount of inorganic mercury in the locus ceruleus, so other reasons for the variable uptake of this toxic metal remain to be found. One possibility is that toxins are taken up by a stressor-activated locus ceruleus.
